# Antioxidant effect of aqueous extract of four plants with therapeutic potential on gynecological diseases; *Semen persicae*, *Leonurus cardiaca*, *Hedyotis diffusa*, and *Curcuma zedoaria*

**DOI:** 10.1186/s40001-017-0293-6

**Published:** 2017-11-25

**Authors:** Shaojian Ji, Amir Fattahi, Nathalie Raffel, Inge Hoffmann, Matthias W. Beckmann, Ralf Dittrich, Michael Schrauder

**Affiliations:** OB/GYN, University Hospital Erlangen, Friedrich-Alexander University, Universitätsstrasse 21-23, Erlangen-Nürnberg, 91054 Erlangen, Germany

**Keywords:** Antioxidants, *Curcuma zedoaria*, Herbal medicine, *Hedyotis diffusa*, *Leonurus cardiac*, *Semen persicae*

## Abstract

**Background:**

Little information is available concerning antioxidant effects of plant teas (water boiled) which are used more commonly in traditional Chinese medicine than other extracts. Thus, we addressed this issue by evaluating the ability of teas from four different plants with therapeutic potential on gynecological diseases.

**Methods:**

The aqueous extracts of *Semen persicae*, *Leonurus cardiaca*, *Hedyotis diffusa,* and *Curcuma zedoaria* rhizome were prepared and then their effects on copper-induced low-density lipoprotein cholesterol (LDL-C) oxidation were evaluated by spectrophotometric method. Density gradient ultracentrifugation method was recruited to isolate LDL-C from healthy individuals.

**Results:**

Our results showed that adding 10, 20, and 30 µl *S. persicae* could increase the lag phase duration of LDL-C oxidation compared with control reaction 12, 21, and 33%, respectively. The most effective delay (87%) was observed when 30 µl *H. diffusa* was added to the reaction. In cases of *L. cardiaca* and *C. zedoaria*, we found no significant influence on the lag phase duration (*p* > 0.05). Moreover, our findings about starting point of the decomposition phase were almost in parallel with the lag phase results, as 30 µl of *S. persicae* or *H. diffusa* teas could significantly increase the initiation time of decomposition (*p* < 0.05).

**Conclusions:**

In conclusion our results showed that both *S. persicae* and *H. di*ffusa teas and not *L. cardiaca* and *C. zedoaria* could have medicinal therapeutic effects partly through direct oxidation prevention.

## Background

Oxidative stress, an imbalance between formation and elimination of reactive oxygen species (ROS), is one of the major causes of many diseases including cancer [1], diabetes mellitus [2], atherosclerosis [3], and more importantly gynecological diseases especially endometriosis [4], polycystic ovarian syndrome (PCOS) [5], and menstruation disturbances [6, 7]. Such negative effects of excessive ROS are due to oxidative damage of various cellular components including lipids, proteins, and nucleic acids [8]. Previous studies have mentioned the beneficial and preventive effects of antioxidant substances on oxidative stress-induced diseases via scavenging free radicals [9, 10]. In this concept, therapeutic effects of various antioxidants on female reproductive system diseases such as dysmenorrhea [11, 12], PCOS [13], and endometriosis [14] have been documented. Although, artificial antioxidants are extensively used in food products and medications but due to their instability and also possible potential in acting as carcinogens, there is a growing interest towards using natural antioxidants [15, 16]. In this case, herbal medicinal plants which are well-known in folk medicine and contain potent antioxidant substances such as phenolic acid, flavonoids, and tannins have attracted a considerable attention [17, 18]. Thus, numerous studies have been conducted to investigate therapeutic and preventive roles of various herbal extracts in gynecological diseases. For example, in a study conducted by Yang et al. [19] beneficial influence of *Panax Ginseng* Meyer on gynecologic complaints including menstrual irregularity and pain through improving oxidative status has been indicated. However, little information is available concerning antioxidant capacity of some herbal plants of which aqueous extractions are used as teas in traditional Chinese medicine; their medicinal potential in female health has been emphasized.

The *Semen persicae*, also called Taoren (or peach kernel), consists of the substantial part of the harvested peaches which is mostly considered as waste and low-value residue [20]. However, the *S. persicae* is considered as one of the important herbal products in traditional Chinese medicines [21]. Its beneficial effects on various pathological situations has been indicated including diverse womb as well as abdominal disorders and most commonly blood stasis [22]. Besides, its lipid and Malondialdehyde (MDA) lowering properties have been documented [23]. Moreover, based on the ancient literature this plant could regulate menstruation and relieve pain (reviewed in Ref. [21]). However, based on our knowledge its possible antioxidant potency especially as aqueous extract has not been investigated yet.

*Leonurus cardiaca* also commonly called motherwort can be found widely throughout Europe and Asia [24]. The *L. cardiaca* has a 100-year history of use for the treatment of nervous and functional cardiac disorders in Europe [25]. Moreover, for many years, traditional Chinese medicines have applied this plant to treat different disorders such as blood stasis, irregular menstrual cycle, urine excretion problems, and inflammatory diseases [26]. Besides, traditional application of this plant in menopause and menstrual disorders (dysmenorrhea or absent menstruation) has been reported [27, 28]. Further researches also confirmed its antinociceptive, hypoglycemic, anticancer, antibacterial, antifungal, and antioxidant properties [29, 30]. However, the antioxidant capacity of its tea has not been sufficiently addressed yet.

*Hedyotis diffusa Willd* (*Oldenlandia diffusa*, Rubiaceae) is an ancient Chinese herbal which can be found in Asian countries including China; its aqueous extract (boiled in water) is employed to treat many kinds of diseases [31]. Applying of the *H. diffusa* for treatment of diverse types of inflammation and cancer have been reported previously [32]. Besides, investigations have shown antifungal, anti-inflammatory, immunoregulatory, and antioxidant attributes of this plant [33] that make this plant a potential therapeutic herbal for female-specific disorders; however, based on our knowledge there is no study about therapeutic application of *H. diffusa* in female reproductive system diseases. Huan-huan et al. [34] demonstrated that alcohol-ethyl acetate extract of this plant has radical scavenging activity and could protect DNA from hydroxyl radical-induced damages. However, antioxidant potency of the *H. diffusa* tea is still unknown.

*Curcuma zedoaria* (Rosc. Zingiberaceae), a plant found in tropical countries, is widely used in the traditional Chinese medicine, especially its tea that is made from dried rhizomes [35]. This plant is clinically applied in treatment of cancers, stomach diseases, chronic pelvic inflammation, blood stagnation, coronary heart disease, and anemia [36]. Powder of the dried plant is also applied for treatment of menstrual irregularities [37]. Previous studies have confirmed its antimicrobial, anti-inflammatory, and analgesic activities [38]. More interestingly, there are evidences about effective antioxidant capacity of the *C. zedoaria* rhizomes essential oil as well as aqueous and methanolic extracts [38, 39].

Considering pivotal roles of oxidative stress in various disorders such as gynecological diseases there is wide interest to use herbal medicinal plants which contain antioxidant substances to prevent such oxidation. On the other hand, most studies have paid attention on oils or other extracts of the plants and little information is available concerning antioxidant effect of the plant teas (water boiled) which are used more commonly than other extracts. Thus, we addressed this issue by evaluating the ability of teas from four different plants to prevent copper-catalyzed low-density lipoprotein cholesterol (LDL-C) oxidation; in particular, we investigated antioxidant activity of four plants with therapeutic potential on gynecological diseases, *S. persicae*, *L. cardiaca*, *H. diffusa*, and *C. zedoaria* tea.

## Methods

### LDL-C fraction isolation, purification, and measurement

Fasting blood samples were collected from healthy volunteers into EDTA-containing tubes and subsequent plasma separation was performed by centrifugation. LDL-C isolation was carried out immediately using density gradient ultracentrifugation. We applied the Abbey et al. [40] protocol briefly as follows: Firstly, using KBr, the plasma density was adjusted to 1.21 g/ml and then the plasma was layered under NaCl solution (*d* = 1.006 g/ml) containing 0.1% EDTA in Quickseal tubes. After preparation of the tubes, they were centrifuged at 4 °C and 280,000×*g* for 6 h by sequential ultracentrifugation (Beckman Ultracentrifuge, type L5-75, Ti 75 rotor). Afterwards, the LDL-C band (yellow band) was collected.

In order to purify the LDL-C particles we applied the gel filtration method. Briefly, the gel bed column was washed twice with PBS buffer and then 800 μl of isolated LDL-C were added to the column; afterwards, the LDL-C was eluted from the column by adding 2.6 ml PBS buffer. To confirm purity of isolated fractions, agar-agarose gel electrophoresis was employed; the results showed that the LDL-C content was more than 96% in all eluates.

LDL-C cholesterol levels were also evaluated in each eluate based on an enzymatic colorimetric method using automatic analyzer (Olympus AU 2700, NY, USA).

### Preparation of the teas

Dried forms of *S. persicae*, *L. cardiaca*, *H. diffusa*, as well as *C. zedoaria* rhizome were purchased from a traditional Chinese pharmacy (Wenzhou, China). Considering that these herbal plants commonly are used as tea through boiling in water, we also prepared the teas with this method. For this purpose, 20 g of each dried plants were added into 100 ml distilled water and the water was heated for 20 to 30 min until it started to boil. After 20 min of boiling, the aqueous extracts were filtered to avoid any solid particles; we let the teas cool down to room temperature and used them freshly for antioxidant evaluation.

### Evaluation of LDL-c oxidation delay

The copper-induced LDL-C oxidation is accompanied by an increase in levels of dienes which have a maximum absorbance at 234 nm. The LDL-C oxidation consists of three phases including lag, propagation, and decomposition phase [41]. The lag phase is an oxidation-resistant step in which diene levels and consequently absorbance at 234 nm is almost constant. However, upon the propagation phase, the absorbance quickly increases and finally reaches the maximum level.

**Fig. 1:**
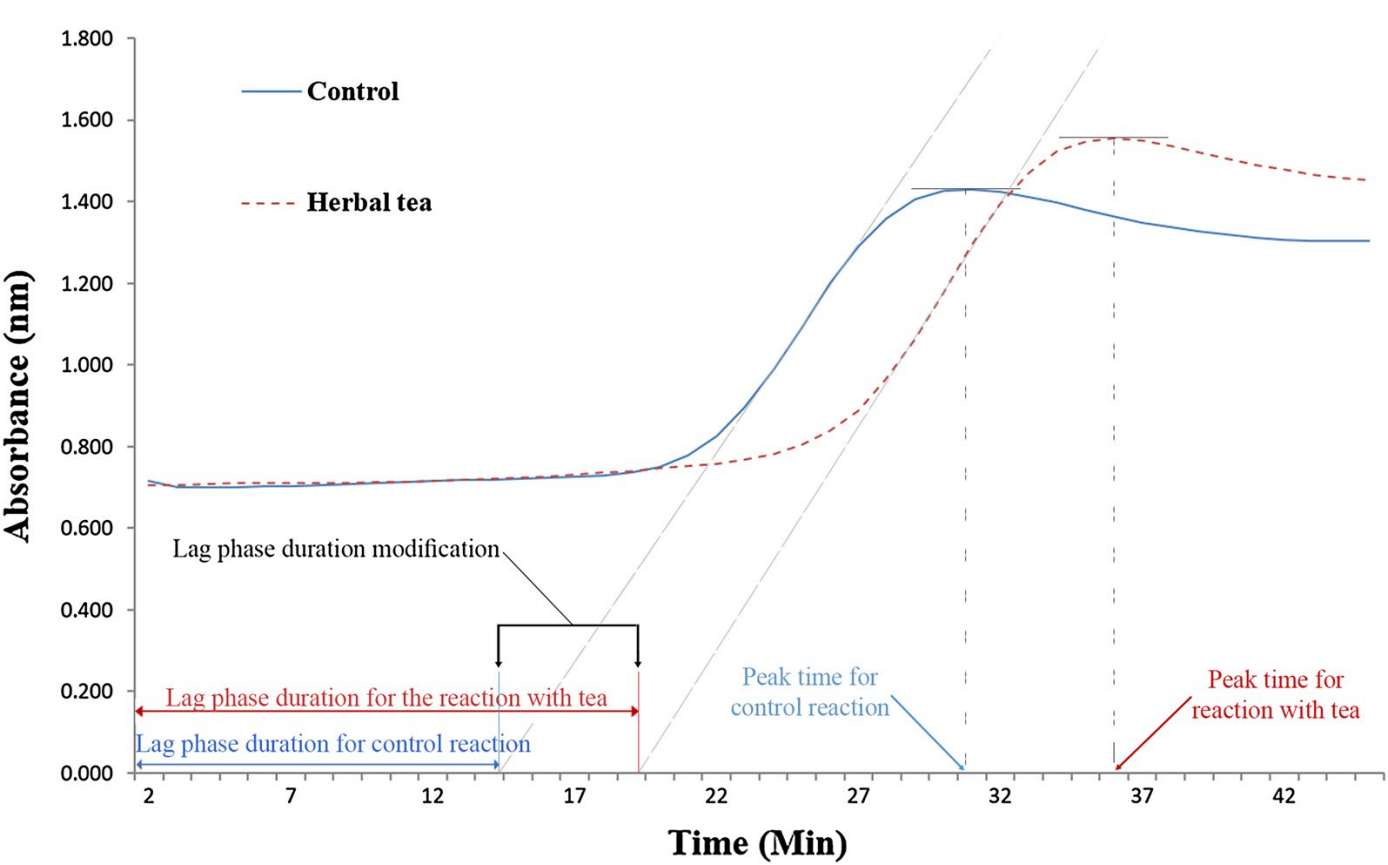
An example for kinetics of LDL oxidation in control and herbal tea added reactions

In the present study we evaluated direct effects of different tea concentrations on preventing LDL-C oxidation and delaying the lag phase duration. For this purpose, 10, 20, and 30 µl of each tea were added into a 0.08 mg LDL-C-containing eluate and the final reaction volume was adjusted to 1 ml using O_2_-saturated PBS buffer. Adding CuSO_4_ to the reaction and mixing the reaction batch initiated the oxidation process; absorbance at 234 nm was continuously measured at 30 °C for maximum 2 h (depending on the lag phase duration). Two reactions without tea (control) and two additional reactions for all three tea concentrations (10, 20, and 30 µl) were carried out for each tea simultaneously. Besides, all reactions were conducted three times. The intercept of the baseline and slope of the absorbance curve in the propagation phase was defined as the lag phase; the data were expressed in minutes. The peak time was considered as the time of maximum absorbance (Fig. 1).

## Statistical analysis

Kolmogorov–Smirnov test was used to confirm the normal distribution of data. One-Way ANOVA test following Tukey’s post hoc test was applied to compare the data among groups. All Statistical analyses were conducted using SPSS (version 16). Differences between means were considered significant when *p* value was 0.05 or less.

## Results

The effects of *S. persicae*, *L. cardiaca*, *H. diffusa*, and *C. zedoaria* teas with various doses (0, 10, 20, and 30 µl) on duration of LDL-C oxidation lag phase are shown in Table 1. Our results showed that adding 30 µl *S. persicae* tea to the LDL-C oxidation reaction could significantly delay the lag phase in comparison to the control reaction without tea (*p* = 0.018). The lag phase durations in lower doses of *S. persicae* tea (10 and 20 µl) were also higher than the control (without tea); nevertheless, this difference did not reach statistical significance (*p* > 0.05). In case of *H. diffusa* tea, we also observed a dose-dependent correlation between tea amount and lag phase duration. As adding 20 µl of the tea to the reaction could cause 29.63 ± 2.12 min lag phase which was significantly higher than lag phase duration in reactions without or with 10 µl of the tea (*p* = 0.001 and 0.023, respectively). Moreover, we found that the lag phase duration in reactions with 30 µl *H. diffusa* tea was statistically significant longer than the reactions without or with 10 and 20 µl of the same tea (*p* < 0.001, *p* < 0.002 and *p* = 0.032, respectively).

**Table 1:** Effect of four herbal teas on the lag phase duration (min) of copper-induced low-density lipoprotein cholesterol oxidation

	Tea concentrations			
	0 (control)	10 µl	20 µl	30 µl
*Semen persicae*	23.75 ± 2.18	26.55 ± 1.83	28.60 ± 5.04	31.37 ± 1.70^a^
*Leonurus cardiaca*	23.20 ± 2.54	25.10 ± 3.67	26.70 ± 3.95	27.30 ± 5.23
*Hedyotis diffusa*	19.06 ± 0.11	23.16 ± 1.04	29.63 ± 2.12^a,b^	35.70 ± 3.48^a,b,c^
*Curcuma zedoaria*	19.75 ± 3.60	22.30 ± 2.12	24.80 ± 3.95	25.70 ± 0.28

Significant difference (*p* < 0.05) in comparison with ^a^Control, ^b^10 µl and ^c^20 µl tea concentrations

**Fig. 2:**
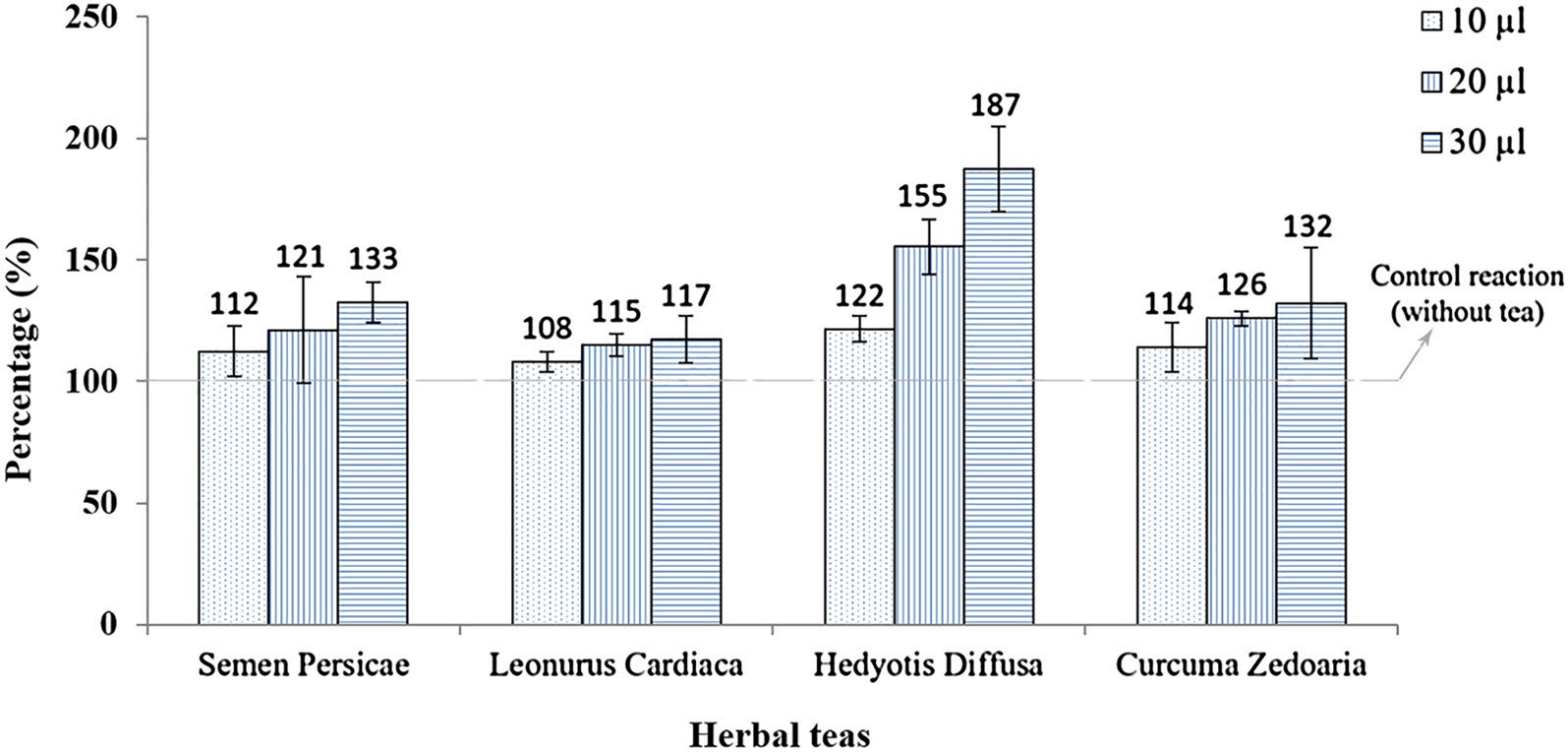
Percentage of the lag phase duration modification in low-density lipoprotein cholesterol oxidation following addition of four teas. All three different quantities per tea are depicted and the control reaction is set as 100%

In order to provide better understanding about effects of the teas on LDL-C oxidation we calculated the lag phase modification of each tea in comparison to corresponding reactions without the tea and illustrated the results as percentage in Fig. 2. We found that higher amounts of the teas (30 µl) could have a higher effect on duration of the lag phase as adding 10, 20, and 30 µl *S. persicae* could increase the phase duration compared with control reaction, respectively, 12, 21, and 33%. The most effective delay was observed when 30 µl *H. diffusa* was added to the reaction; it caused about 87% increase in the lag phase duration. Adding 10 and 20 µl of *H. diffusa* caused a lag phase extension of 22 and 55%, respectively. We further evaluated peak time in all reactions; peak time shows the starting point of the decomposition phase in LDL-C oxidation (Table 2). The obtained results were almost in parallel with our findings about lag phase, as addition of 30 µl *S. persicae* and *H. diffusa* tea could significantly increase the peak time compared to reactions without and with 10 µl of the teas. (for *S. persicae p* = 0.001 and 0.016 and for *H. diffusa p* = 0.003 and 0.009, respectively).

**Table 2:** Comparison of the peak time (min) of copper-induced low-density lipoprotein cholesterol oxidation among reactions with four herbal teas

	Tea concentrations			
	0 (control)	10 µl	20 µl	30 µl
*Semen persicae*	33.75 ± 2.21	37.75 ± 2.36	41.50 ± 5.68	47.75 ± 4.27^a,b^
*Leonurus cardiaca*	34.0 ± 1.41	36.50 ± 3.53	38.00 ± 4.24	40.00 ± 8.48
*Hedyotis diffusa*	33.33 ± 0.57	36.66 ± 0.57	44.00 ± 4.00	53.00 ± 8.00^a,b^
*Curcuma zedoaria*	33.00 ± 4.24	35.50 ± 3.53	38.50 ± 4.94	41.00 ± 2.83

Significant difference (*p* < 0.05) in comparison with ^a^Control and ^b^10 µl tea concentration

## Discussion

Considering existence of wide interest among people to use various medicinal plants as teas and also due to lack of information about antioxidant effects of such teas, we investigated antioxidant potency of *S. persicae*, *L. cardiaca, H. diffusa*, and *C. zedoaria* teas in preventing LDL-C oxidation in the present study.

In case of *S. persicae*, we found that adding 30 µl of the tea to LDL-C oxidation reaction could prolong the lag phase and prevent oxidation of LDL-C. An ancillary increase in peak time of LDL-C oxidation following the tea adding demonstrated the potential of this tea in even decreasing oxidation rate; however, lack of significant effects with lower amounts (10 and 20 µl) revealed that the antioxidant capacity is not that much potent in these cases. Previous studies on *S. persicae*, have mostly focused on its anti-coagulatory and anti-inflammatory effects; although the latter effect could also be partly a result of its antioxidant activity [42]. However, in accordance with our findings, Wu et al. [43] have reported the ability of *S. persicae* meal in scavenging radicals, notwithstanding they used various chemical solutions such as petroleum ether to extract the meal. Moreover, in an in vivo study, it has been demonstrated that oral administration of *S. persicae* decoction in hyperlipidemia patients could decrease plasma levels of MDA which is a lipid peroxidation marker [44]. Considering the ability of the tea in delaying LDL-C lag phase as well as slowing oxidation propagation phase, it could be postulated that the antioxidant activity of *S. persicae* tea includes chelation of copper ions, neutralizing free radicals, and decomposing ox-LDL-C. Such antioxidant activities could be obtained from phenolic compounds of the tea [45]. Although we did not evaluate phenolic composition of the tea; nevertheless, previous studies showed the existence of high amounts of phenolic compounds in *S. persicae* meals extracted by Soxhlet extractor [43]. So most likely the tea also has such components and could play a role as an antioxidant solution. Higher antioxidant activity of 30 µl tea in comparison to lower amounts in preventing LDL-C oxidation is logical as the higher tea amount would have higher quantities of phenolic compounds and an increased antioxidant activity; this is in agreement with previously reported findings [43, 46]. So it could be postulated that therapeutic effect of *S. persicae* on endometriosis [47], removing blood stasis [22], and menstrual irregularity [21] could be partly through its antioxidant properties, as an association between oxidative stress and these disorders has been previously reported [4, 6, 7].

*H. diffusa*, like *S. persicae*, showed antioxidant activity even in lower amounts. We found that adding 30 µl of the *H. diffusa* tea to the LDL-C oxidation reaction could prolong the lag phase about 87% and also could significantly increase the peak time. Such a strong inhibitory effect of this tea in inhibiting LDL-C oxidation could introduce the *H. diffusa* tea as a potential drink to prevent oxidative stress-related gynecological diseases such as PCOS and endometriosis. However, further in vivo studies on patients with oxidative stress-related diseases are required to clarify the tea beneficial influences. Although the water extract of this plant has not been evaluated in terms of antioxidant activity and just its anticancer properties have been indicated [31], Bhuyan et al. [48] have reported radical scavenging capacity of the plant ethanol extract; this is in confirmation of our study. Besides, Huan-huan and colleagues [34] have documented that alcohol-ethyl acetate extract of *H. diffusa* has the ability to chelate ferrous iron, so one of the possible mechanisms of the tea in preventing copper-induced LDL-C oxidation might be chelating copper ions. Another possible reason for inhibitory effects of *H. diffusa* on LDL-C oxidation could be the presence of phenolic compounds such as phenolic acids and flavonols, as reported previously [49]. Moreover, in a study conducted by Yan et al. [50], four polysaccharides with strong hydroxyl radical scavenging activity in water/alkaline extraction and ethanol precipitation of *H. diffusa* have been detected. Yan et al. also claimed that higher doses from two of the polysaccharides have a more significant radical scavenging activity than vitamin C at the same dose. The presence of such polysaccharides in the tea could also be considered as a reason for its antioxidant capacity.

Interestingly, we found no statistically significant effect of *L. cardiaca* tea on LDL-C oxidation. Contrary to our findings, antioxidant activity of *L. cardiaca* alcoholic extract has been reported in both in vitro and in vivo conditions [51, 52]. Lack of significant effects of *L. cardiaca* tea on LDL-C oxidation in our study could be considered from different points of view. First, it is possible that reported beneficial influences of *L. cardiaca* on oxidative stress and lipid peroxidation are results of indirect effects of the plant rather than the direct mechanism. Supporting this idea, it has been mentioned that the antioxidant effects of *L. cardiaca* are only present in oxidative stress conditions and most probably through increasing antioxidant enzyme activities such as superoxide dismutase (SOD) and glutathione peroxidase (GPx) [53]. However, reports about in vitro antioxidant activity of the plant extract implies potential of a direct radical scavenging ability [30]. Second explanation for our contrary results could be absence or low concentration of antioxidant compounds in *L. cardiaca* plant; however, reports about presence of antioxidant compounds such as flavonoids, triterpenoids, and alkaloids in the plant extracts rejects such a hypothesis [54]. Another possible reason could be inadequacy of water as a solvent for preparing *L. cardiaca* extract with antioxidant capacity. In support of this reason, it has been reported that the water extract of *L. cardiaca* fruits had lower antioxidant activity compared with ethyl acetate, propyl alcohol, and supercritical CO_2_ extracts [55]. Thus, adding higher amounts of water-boiled extract (tea) might have caused a statistically significant effect on LDL-C oxidation. However, it should be mentioned that our aim was to evaluate antioxidant capacity of herbal medicine prepared using traditional Chinese medicine method rather than lipophilic extraction methods. Another possible reason could be inadequacy of water as a solvent for preparing *L. cardiaca* extract with antioxidant capacity. In support of this reason, it has been reported that the water extract of *L. cardiaca* fruits had lower antioxidant activity compared with ethyl acetate, propyl alcohol, and supercritical CO_2_ extracts [55]. Thus, adding higher amounts of water-boiled extract (tea) might have caused a statistically significant effect on LDL oxidation. A further potential reason for lack of antioxidant capacity in this plant could be due to the thermal condition in the tea preparation. However, this possibility seems unlikely because the plant was boiling for up to 20 min and the time duration is not comparable with the studies that have reported adverse effects of heating [56]. On the other hand, some studies even reported beneficial effect of such a short heating for increasing antioxidant activity of the herbal extracts [57, 58].

Like *L. cardiaca* tea, the tea prepared from *C. zedoaria* also did not have a significant influence on LDL-C oxidation which is in contradiction with previous findings. Previous studies have indicated antioxidant activity of *C. zedoaria* essential oil [38]. Also it has been reported that *C. zedoaria* extracts, prepared using methanol, chloroform, hexane, and ethyl acetate, could have various antioxidant activities such as scavenging and chelating abilities [39, 59]. The abovementioned reasons for *L. cardiaca* could also be considered as explanations for contradictory results in case of *L. cardiaca* tea.

## Conclusions

In conclusion, our results show that both *S. persicae* and *H. diffusa* teas could prevent and also decrease the rate of LDL-C oxidation. However, statistically significant effects were not found for the *L. cardiaca* and *C. zedoaria* teas, possibly due to an inefficient extracting method and/or low antioxidant capacity of the plants. Considering the high prevalence of consuming medicinal plants as teas and on the other hand necessity of evaluating potential antioxidant capacity as well as side effects of such teas, future studies should shed more light on these issues; including beneficial effects in oxidative stress-related gynecological diseases.

## Abbreviations

MDA: malondialdehyde
PCOS: polycystic ovarian syndrome
ROS: reactive oxygen species
SOD: superoxide dismutase
GPx: glutathione peroxidase
